# Evaluation of inhomogeneity correction factors for 6 MV flattening filter‐free beams with brass compensators

**DOI:** 10.1120/jacmp.v14i3.3990

**Published:** 2013-05-06

**Authors:** Joshua Robinson, Daniel Opp, Geoffrey Zhang, Vladimir Feygelman

**Affiliations:** ^1^ Department of Radiation Oncology Moffitt Cancer Center Tampa FL; ^2^ Department of Physics University of South Florida Tampa FL USA

**Keywords:** flattening filter‐free, compensator, inhomogeneity correction factor, dose calculation accuracy, lung

## Abstract

The 6 MV flattening filter‐free (FFF) beam has been commissioned for use with compensators at our institution. This novel combination promises advantages in mitigating tumor motion due to the reduced treatment time made possible by the greatly increased dose rate of the FFF beam. Given the different energy spectrum of the FFF beam and the beam hardening effect of the compensator, the accuracy of the treatment planning system (TPS) model in the presence of low‐density heterogeneities cannot be assumed. Therefore, inhomogeneity correction factors (ICF) for an FFF beam attenuated by brass slabs were measured and compared to the TPS calculations in this work. The ICF is the ratio of the point dose in the presence of inhomogeneity to the dose in the same point in a homogeneous medium. The ICFs were measured with an ion chamber at a number of points in a flat water‐equivalent slab phantom containing a 7.5 cm deep heterogeneity (air or $0.27\;g/{\text{cm}}^{3}$ wood). Comparisons for the FFF beam were carried out for the field sizes from 5×5 to $20\times 20\;{\text{cm}}^{2}$ with the brass slabs ranging from 0 to 5 cm in thickness. For a low‐density wood heterogeneity in a slab phantom, with the exception of the point 1 cm beyond the proximal buildup interface, the TPS handles the inhomogeneity correction with the brass‐filtered 6 MV FFF beam at the requisite 2% error level. The combinations of field sizes and compensator thicknesses when the error exceeds 2% (2.6% maximum) are not likely to be experienced in clinical practice. In terms of heterogeneity corrections, the beam model is adequate for clinical use.

PACS number: 87.56.ng

## I. INTRODUCTION

Flattening filter‐free (FFF) beams produce a higher dose rate than conventional beams.^(1)^ The resulting shortened delivery times could be advantageous, among other things, for tumor motion management. For example, if respiratory gating is used, increased dose rate should allow a beam to be delivered over fewer breathing cycles, thus improving delivery efficiency. If the beam on time is short enough, breath‐hold delivery becomes feasible. However, when MLC‐based intensity modulation is used, mechanical constraints often prevent the beam on time from decreasing as a straight reciprocal of the dose rate. More importantly, interplay between the segment sequencing and tumor motion can affect the target dose coverage.^2^, ^3^ Another option for delivering IMRT is solid compensators.^(4)^ While still offering the benefits of inverse planning, this technique is more robust in terms of the tumor motion effect on dosimetry.^3^, ^5^, ^6^ It was recently shown^(7)^ that compensator‐based IMRT can be commissioned for the 6X FFF beam with sufficient accuracy in a homogeneous phantom, and thus could be used clinically for example for abdominal tumors. However, compensator‐based IMRT could be of interest for moving targets surrounded by a large volume of low‐density tissue, such as in lung or esophageal cancers. The collapsed cone convolution (CCC) algorithm as implemented in Pinnacle treatment planning system (TPS) historically has proven to be sufficiently accurate under these conditions.^(8)^ However, the energy spectra of the FFF beams differ substantially from the conventional ones because the low‐energy photons are no longer removed by the (absent) flattening filter. It cannot be a priori assumed that after propagating this new type of a beam through a brass filter, the TPS would maintain the same accuracy of dose calculations in a low‐density media. Therefore, we set out in this paper to evaluate the accuracy of the inhomogeneity corrections for the commercial dose calculation algorithm when a 6X FFF beam is used with the brass filters. Since the CCC algorithm was extensively studied before, the goal of this Technical Note is to generate the basic benchmark data similar to that presented in the tables in the AAPM TG65 Report, as opposed to the comprehensive evaluation of the algorithm. The data for a conventional 6X beam collected under the same conditions are presented as a benchmark. In addition, a special case of a cylindrical phantom with a large internal air cavity corresponding to commercial diode array dosimeter geometry was studied. In the past, this geometry proved to be a good discriminator between the different algorithms’ ability to model large low‐density inhomogeneities.^(9)^

## II. MATERIALS AND METHODS

A TrueBeam linac (Varian Medical Systems, Palo Alto, CA) operating in 6X FFF (1400 MU/min) and standard 6X beam (600 MU/min) modes was used. Pinnacle TPS v 9.2 (Phillips Radiation Oncology, Fitchburg, WI) was used for dose calculations.

**Figure 1:**
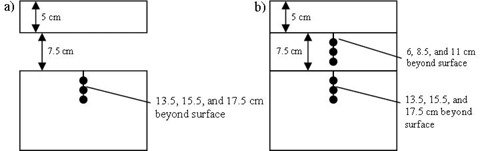
The slab phantom geometry and points of interest: (a) the air inhomogeneity setup contains points at 13.5, 15.5, and 17.5 cm depth; (b) The $0.27\;g/{\text{cm}}^{3}$ wood inhomogeneity contains additional points at 6, 8.5, and 11 cm depth.

Measurements were made with air and low‐density wood $\left(0.27\;g/{\text{cm}}^{3}\right)$ inhomogeneities in a water‐equivalent $30\times 30\;{\text{cm}}^{2}$ slab phantom (Plastic Water, CIRS Inc., Norfolk, VA). The general phantom configuration was 5 cm of water‐equivalent material followed by a 7.5 cm inhomogeneity, and finally 16.5 cm of backscatter material (Fig. 1(a)). The inhomogeneity correction factor (ICF) is the ratio of the point dose in the presence of inhomogeneity to the dose in the same point in a homogeneous medium.^(8)^

(1) $$ICF=\left(\frac{\text{point}\;\text{dose}\;\text{with}\;\text{inhomogeneity}}{\text{dose}\;\text{at}\;\text{same}\;\text{point}\;\text{in}\;\text{homogeneons}\;\text{medium}}\right)$$

**Figure 2:**
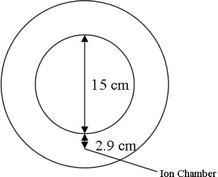
Cross‐section of the cylindrical phantom simulating the ArcCHECK geometry. The 15 cm cavity can be plugged with a PMMA cylinder to render the phantom homogeneous. The location of the exit point of interest 2.9 cm from the edge of the inhomogeneity where the ion chamber was placed is shown.

The measured and calculated ICFs were compared. All measurements were made with a 0.06 cc A1SL ion chamber (Standard Imaging Inc., Middleton, WI). The ion recombination correction exhibited negligible change (0.1%) in the encountered range of dose rates. Since all the measurements were acquired in the same session, raw chamber readings were used to determine the ICF. For both the air and wood inhomogeneities, the points of interest were selected 13.5, 15.5, and 17.5 cm below the surface (Fig. 1(a)). The low‐density wood slab was also drilled to accommodate the same ion chamber and additional points of interest were chosen inside the heterogeneity, 6, 8.5, and 11 cm below the phantom surface (Fig. 1(b)). For each point, data were collected with the 6X FFF beam for 5×5,10×10, and $20\times 20\;{\text{cm}}^{2}$ field sizes with brass filters 0, 1, 2, 3, and 5 cm thick. The brass slabs mounted on plastic trays were placed in the first accessory slot on the linac. The CCC algorithm's ICF comparison to measurement for a standard open 6X beam is well‐documented. In addition to the standard slab phantom ICF evaluation, a special case was studied that represented the geometry of a cylindrical diode array used for IMRT QA (ArcCHECK, Sun Nuclear Corp., Melbourne, FL).^(9)^ In its standard configuration, that phantom is a hollow cylinder with an air cavity 15.1 cm in diameter. A PMMA shell mimicking the dosimeter was constructed. The shell was drilled to accommodate an ion chamber at certain diode locations (2.9 cm inside the surface of the shell, see Fig. (2)). The plastic shell with an ion chamber was used instead of the actual ArcCHECK to avoid the variation in the diodes’ response with changing scattering conditions due to the diodes’ energy dependence. A PMMA plug can be inserted into the phantom rendering it a homogeneous cylinder to derive the ICF. For these experiments the same field sizes from 5×5 to $20\times 20\;{\text{cm}}^{2}$ were used, and the brass filter thickness varied from 1 to 3 cm.

## III. RESULTS

**Figure 3:**
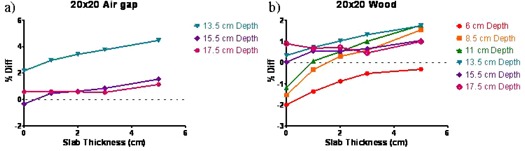
Percent difference between calculated and measured ICF vs. brass slab thickness for the $20\times 20\;{\text{cm}}^{2}$ field in the flat phantom: (a) air inhomogeneity; (b) wood inhomogeneity.

The differences between the measured and calculated ICFs as a function of the brass slab thickness are plotted for a large field in Figs. 3 (a) and (b), for the air and wood inhomogeneities, respectively. With the 7.5 cm air gap in a slab phantom and points ≥3 cm beyond the interface, the largest difference between the calculated and measured 6X FFF ICF was 1.5% (Fig. 3(a)). The ICF differences exceeded 2% only for the point 1 cm beyond the air gap. The error increased with field size and brass thickness and reached 4.5% for the $20\times 20\;{\text{cm}}^{2}$ field with the 5 cm thick brass slab. Table 1 shows that, at this point, the open standard 6X beam also exhibited disagreement between the calculated and measured ICF, up to 3.4%.

The results, similar to Fig 3 but for the smaller field sizes, are presented in 4, 5. With the low‐density wood, the 6X FFF ICF difference exceeded 2% only for the smaller field sizes $\left(\leq 10\times 10\;{\text{cm}}^{2}\right)$, with thin brass (0 to 1 cm), and only for the point 1 cm beyond the proximal buildup interface (4, 5). The maximum ICF deviation was −2.6% for the $5\times 5\;{\text{cm}}^{2}$ open 6X FFF beam, compared to −2.1% for the conventional one (see Table 2). The vast majority of the points exhibit ICF agreement between measurement and calculation better than 2%.

**Table 1:** ICF percent difference (measured‐TPS) for 6X open beam at selected points on the flat air gap phantom.

*7.5 cm Air Inhomogeneity*		
*6X*		
*13.5 cm Below Surface*		
$5\times 5\;{\text{cm}}^{2}$	$10\times 10\;{\text{cm}}^{2}$	$20\times 20\;{\text{cm}}^{2}$
3.1%	3.4%	3.2%
*15.5 cm Below Surface*		
$5\times 5\;{\text{cm}}^{2}$	$10\times 10\;{\text{cm}}^{2}$	$20\times 20\;{\text{cm}}^{2}$
−0.7%	−0.5%	−0.3%
*17.5 cm Below Surface*		
$5\times 5\;{\text{cm}}^{2}$	$10\times 10\;{\text{cm}}^{2}$	$20\times 20\;{\text{cm}}^{2}$
−0.8%	−0.4%	0.7%

**Figure 4:**
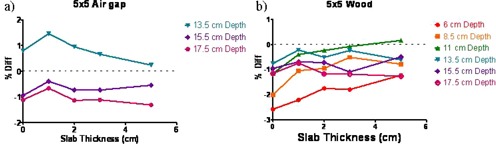
Percent difference between calculated and measured ICF vs. brass slab thickness for the $5\times 5\;{\text{cm}}^{2}$ field in the flat phantom: (a) air inhomogeneity; (b) wood inhomogeneity.

Table 3 summarizes the results for the TPS vs. measured ICF comparisons for the ArcCHECK exit detector.

The errors ranged from −3.0% to 0.3% over the range of field sizes and brass slab thicknesses for the 6X FFF beam. The conventional 6X open beam error did not exceed 1.1%.

**Figure 5:**
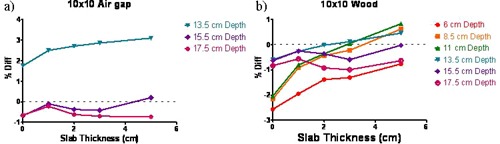
Percent difference between calculated and measured ICF vs. brass slab thickness for the $10\times 10\;{\text{cm}}^{2}$ field in the flat phantom: (a) air inhomogeneity; (b) wood inhomogeneity.

**Table 2:** ICF percent difference (measured‐TPS) for 6X open beam at selected points on the flat wood phantom.

*7.5 cm Wood Inhomogeneity*		
*6X*		
*6 cm Below Surface*		
$5\times 5\;{\text{cm}}^{2}$	$10\times 10\;{\text{cm}}^{2}$	$20\times 20\;{\text{cm}}^{2}$
−2.1%	−0.6%	−1.3%
*8.5 cm Below Surface*		
$5\times 5\;{\text{cm}}^{2}$	$10\times 10\;{\text{cm}}^{2}$	$20\times 20\;{\text{cm}}^{2}$
−0.3%	−1.4%	−1.0%
*11 cm Below Surface*		
$5\times 5\;{\text{cm}}^{2}$	$10\times 10\;{\text{cm}}^{2}$	$20\times 20\;{\text{cm}}^{2}$
0.4%	−1.6%	−0.9%
*13.5 cm Below Surface*		
$5\times 5\;{\text{cm}}^{2}$	$10\times 10\;{\text{cm}}^{2}$	$20\times 20\;{\text{cm}}^{2}$
−0.2%	0.0%	0.9%
*15.5 cm Below Surface*		
$5\times 5\;{\text{cm}}^{2}$	$10\times 10\;{\text{cm}}^{2}$	$20\times 20\;{\text{cm}}^{2}$
−0.3%	−0.1%	0.4%
*17.5 cm Below Surface*		
$5\times 5\;{\text{cm}}^{2}$	$10\times 10\;{\text{cm}}^{2}$	$20\times 20\;{\text{cm}}^{2}$
−0.6%	−0.3%	1.3%

**Table 3:** ICF percent difference (ion chamber measurement‐TPS) at the exit ArcCHECK detector location.

*ArcCHECK ICF at Exit*						
*6X FFF*					*6X*	
*Brass Thickness (cm)*	$5\times 5\;{\text{cm}}^{2}$	$10\times 10\;{\text{cm}}^{2}$	$20\times 20\;{\text{cm}}^{2}$	$5\times 5\;{\text{cm}}^{2}$	$10\times 10\;{\text{cm}}^{2}$	$20\times 20\;{\text{cm}}^{2}$
0	−2.4%	−1.3%	0.3%	−1.0%	−0.5%	1.1%
1	−2.3%	−1.3%	0.3%			
2	−2.7%	−1.6%	0.3%			
3	−3.0%	−1.9%	0.5%			

The experimental setup had a combined uncertainty of ∼0.4%. The sources of uncertainty added in quadrature were setup error, dose calculation error due to uncertainty in the material, and systematic repeatability of the measurements with associated errors of 0.4%, 0.1%, and 0.1%, respectively.

## IV. DISCUSSION & CONCLUSION

With the exception of the point 1 cm beyond the 7.5 cm air gap, the TPS handles the inhomogeneity correction with the brass‐filtered 6X FFF beam at the requisite 2% level^(8)^ in the slab phantom. The larger error at a point 1 cm beyond the sizable air gap is understandable as electronic equilibrium is likely not yet reestablished there. Inside and beyond the lung‐type heterogeneity, the measured ICFs generally agree with calculations within 2%, save a few points. In our beam model, the largest error (−2.6%) occurred for an open beam, which is least relevant in the context of compensator‐based IMRT. The general trend for the difference between measured and calculated ICFs is overcorrection on the part of the TPS for the small field size to undercorrection for the large field size — a trend that is reflected in the standard 6X beam, as well. The general trend from over‐ to undercorrection can also be observed with the increase in brass thickness. This trend diminishes with depth, but persists deeper with the larger field sizes. This relationship to the field size indicates that modeling the scatter from the compensator is the likely driving factor behind this trend.

The inhomogeneity‐related errors introduced by a large air cavity in the ArcCHECK phantom should have only modest effect on agreement between the measured and calculated dose distributions. The largest ICF errors of 3% are limited to the small field sizes, which we have not experienced clinically, so far. But even in this extreme case, the contribution of this error to the overall sum of the entrance and exit dose is not expected to exceed 1%.^(9)^

Our comparison of ICFs in the lung‐density phantom indicates that the 6X FFF beam with compensators is modeled in Pinnacle adequately in terms of the magnitude of dose errors introduced by a low‐density heterogeneity. The TPS model can be used for treating tumors surrounded by lung tissue.

## References

[1] Georg D , Knoos T , McClean B . Current status and future perspective of flattening filter free photon beams. Med Phys. 2011;38(3):1280–93. 21520840 10.1118/1.3554643

[2] Keall PJ , Mageras GS , Balter JM , et al. The management of respiratory motion in radiation oncology report of AAPM Task Group 76. Med. Phys. 2006;33(10):3874–900. 10.1118/1.2349696 17089851

[3] Court LE , Wagar M , Ionascu D , Berbeco R , Chin L . Management of the interplay effect when using dynamic MLC sequences to treat moving targets. Med Phys. 2008;35(5):1926–31. 18561668 10.1118/1.2896083

[4] Jiang SB and Ayyangar KM . On compensator design for photon beam intensity‐modulated conformal therapy. Med Phys. 1998;25(5):668–75. 9608477 10.1118/1.598250

[5] 5. Bortfeld T, Jokivarsi K , Goitein M , Kung J , Jiang SB . Effects of intra‐fraction motion on IMRT dose delivery: statistical analysis and simulation. Phys Med Biol. 2002;47(13):2203–20. 12164582 10.1088/0031-9155/47/13/302

[6] Ehler E , Kim Y , Arvidson N , Nelms B , Tome W . On the dose delivered to a moving target while employing different IMRT delivery mechanisms [abstract]. Med Phys. 2006;33(6):2296.

[7] Robinson J , Opp D , Zhang G , et al. Evaluating dosimetric accuracy of flattening filter free compensator‐based IMRT: measurements with diode arrays. Med Phys. 2012;39(1):342–52. 22225304 10.1118/1.3671936

[8] Papanikolaou N , Battista JJ , Boyer AL , et al. Tissue inhomogeneity corrections for megavoltage photon beams: report of Task Group No. 65 of the Radiation Therapy Committee. AAPM report No. 85. Madison, WI: Medical Physics Publishing; 2004.

[9] Kozelka J , Robinson J , Nelms B , Zhang G , Savitskij D , Feygelman V . Optimizing the accuracy of a helical diode array dosimeter: a comprehensive calibration methodology coupled with a novel virtual inclinometer. Med Phys. 2011;38(9):5021–32. 21978046 10.1118/1.3622823

